# Development and Evaluation of a Candidate Inactivated Vaccine Against Bluetongue Virus Serotype 4 (BTV4)

**DOI:** 10.3390/vaccines12121326

**Published:** 2024-11-26

**Authors:** Ljubisa Veljovic, Dimitrije Glisic, Marko Kirovski, Ljiljana Paušak, Vesna Milicevic

**Affiliations:** 1 Institute of Veterinary Medicine of Serbia, Janisa Janulisa 14, 11000 Belgrade, Serbia; dimitrije.glisic@nivs.rs (D.G.); vesna.milicevic@nivs.rs (V.M.); 2 Veterinarski zavod Subotica, Beogradski put 123, 24000 Subotica, Serbia; marko.kirovski@vetzavod.com (M.K.); ljiljana.pausak@vetzavod.com (L.P.)

**Keywords:** bluetongue virus, inactivated vaccine, candidate vaccine, quality of vaccine, safety, efficacy

## Abstract

Objectives: Although bluetongue is not a contagious disease, it is easily transmitted and spread by appropriate insect vectors, causing great economic damage. Climate change has led to the fact that vectors and diseases have spread to the top of Northern Europe, causing great economic losses in livestock production. An even greater problem is controlling the disease, because numerous species of domestic and wild ruminants are susceptible to bluetongue. The most effective tool against bluetongue disease is vaccination. Methods: Our goal was to carry out laboratory tests of the starting material and the finished product of the candidate inactivated vaccine against BTV4, and to comment on the obtained laboratory results and the results of previously performed clinical studies. There is no ideal vaccine against the bluetongue virus (BTV) due to the serotype diversity of its strains. Thus, there is a need for a vaccine for at least 24 clinically significant serotypes. Sometimes, it is difficult to obtain the desired amount of vaccine against a defined serotype on the market, and this has led to the need for a new vaccine against bluetongue disease. In this study, we tested an experimental inactivated vaccine against BTV4. The master seed BTV4 was examined and characterized by sequencing. Results: The candidate BTV4 vaccine induced the onset of immunity at the latest at day 21 after the application of the first dose in more than 80% of the vaccinated individuals, while the ELISA test detected specific antibodies against BTV for more than a year. Along with our laboratory results, the preliminary results of safety and efficacy trials are also presented. Conclusions: The side effects of this inactivated BTV4 vaccine are within the limits of permissible local reactions without generalized changes in the health status, while the serology and challenge test leads to the conclusion that this vaccine against BTV4 protects a high percentage of vaccinated individuals against BTV4 or causes a significant reduction in the intensity and duration of the clinical signs in the vaccinated sheep. Based on the trial results, the new vaccine has given encouraging results in terms of quality, safety, and preliminary efficacy tests. Thus, we believe that a new vaccine against BTV is on the horizon.

## 1. Introduction

Bluetongue (BT) was first detected at the end of the 19th century, when sheep were imported into South Africa and became infected, showing different clinical signs of hemorrhagic disease [1]. Bluetongue causes great direct and indirect economic damage. The direct economic damages refer to the health condition of the affected individuals, mortality rates, etc., while the indirect damages refer to the prohibition of export and trade. Bluetongue virus (BTV) belongs to the *Orbivirus* genus within the family *Reoviridae* and has a double-stranded RNA genome that encodes four non-structural (NS1–NS4) and seven structural proteins (VP1–VP7) [2]. The viral genome is highly susceptible to reassortment due to its segmentation, and so far, more than 36 serotypes of the BT virus have been described [3]. BTV serotypes 1–24 are classified as being considered significant for animal health. Though BTVs share common group antigens, they can be distinguished by the serotype-specific virus neutralization test (VNT) [4,5] due to there being no cross-reactivity. BTV causes bluetongue disease (BT), which is a highly infectious and non-contagious arthropod vector-borne disease in ruminants worldwide [4]. The virus is mainly transmitted by *Culicoides* spp. Other transmission routes such as oral transmission and transplacental transmission have also been described [4]. The disease affects domestic and wild ruminants, and causes significant economic losses for livestock production [4]. The eradication of the disease is based on the culling of infected animals and vaccination with a serotype-specific vaccine [5]. Live attenuated vaccines are still used today in countries where the use of live vaccines is not prohibited. However, inactivated vaccines against bluetongue disease are used the most due to their safety, but they still carry a lower risk of reversibility. This risk is lower than the risk associated with the use of attenuated vaccines. Attenuated vaccines carry a moderate risk of reverse pathogenicity of the vaccinal virus strain. Recent research has led to the development of the DISA (Disabled Infectious Single Animal) vaccine, which is an attenuated vaccine with the VP3 gene responsible for viremia deleted, but this new generation of live vaccines is not yet in use [6].

BTV4 occurred in Serbia for the first time in 2014, and later on in 2016, when 414 outbreaks were reported [7]. The disease was successfully controlled by implementing strict restrictive measures, stamping out, and vaccination with the monovalent inactivated BTV4 vaccine in infected and high-risk areas determined by the Ministry of Agriculture, Forestry and Water Economy of the Republic of Serbia, Veterinary Directorate. Vaccination was practiced until 2022. The last outbreak of BTV4 was reported in 2020.

Due to the large-scale epidemics and the shortage of vaccines available, the company Veterinarski Zavod Subotica (VZS) developed an inactivated BTV4 vaccine against BTV4. The aim of this study was to investigate the capacity of the candidate BTV vaccine to stimulate immune responses in sheep and to evaluate if these responses could protect sheep from viral challenge. Data supporting the quality of the candidate vaccine are also presented. The preliminary results of this study suggest that the candidate vaccine is safe and efficacious.

## 2. Materials and Methods

The candidate BTV-4 vaccine is an inactivated oil-based formulation that incorporates Montanide ISA 61 (produced by SEPPIC, France) as the adjuvant [8]. The master seed virus (MSV) used in the vaccine, which represents bluetongue serotype 4, was isolated from the blood of infected sheep in the Kraljevo district of Serbia. This MSV was produced using the master cell line (MCL) “BHK 21 clone 13”, sourced from the ATCC animal cell line collection (CCL 10). The composition of one dose of the novel test vaccine is detailed in Table 1.

### 2.1. BTV4 Characterization in MVS

#### 2.1.1. Reverse Transcription Quantitative Real-Time PCR (RT-qPCR)

The lyophilized MSV was reconstituted with 1 mL of distilled water, vortexed, and after complete dissolution, MVS was used for nucleic acid extraction. Viral nucleic acid was extracted using the IndiSpin Pathogen Kit (Indical Bioscience GmbH, Leipzig, Germany). Extraction was carried out using the automatic extraction device QIA cube connect produced by Qiagen (GmbH, Hilden, Germany). Real-time RT-PCR amplification was completed using “Luna Universal Probe One Step RT-PCR” (produced by New England, BioLabs, Ipswich, MA, USA) and previously published primers/probes [9].

#### 2.1.2. Virus Isolation

Virus growth was confirmed by virus isolation on BHK21 clone 13 and subsequent real-time RT-qPCR [10].

Virus isolation was performed by inoculating 100 µL of the master seed virus into a flask of 75 cm^2^ with the 24 h old BHK 21 clone 13 cell line. Another flask was inoculated with a double volume of previously neutralized virus with hyperimmune anti-BTV4 serum. The medium in flasks was changed before inoculation with Dulbecco’s minimum essential medium (DMEM) without the addition of fetal calf serum. The flasks were incubated for 5 days at 37 °C with 5% CO_2_ until the appearance of a clear cytopathogenic effect (CPE) in the flask with the BT virus that was not previously neutralized. The flask with the neutralized BT virus must remain free of cytopathogenic effects and the cells of BHK21 clone 13 cell line remained intact.

#### 2.1.3. Sequencing of Master Seed Strain Bluetongue Virus Serotype 4, Strain 4/LP/2016

##### Virus Propagation

For the propagation of the BTV4 strain, the BHK-21 cell line was used. The strain was inoculated on a confluent monolayer using DMEM (Gibco, Grand Island, NY, USA) supplemented with 5% heat-inactivated fetal bovine serum (Gibco, Grand Island, NY, USA). Additionally, the DMEM was supplemented with a 0.5 mL MycoZap ampule containing a combination of antibiotics and antimetabolic agents, according to the manufacturer’s instructions (Lonza, Basel, Switzerland). The virus was incubated at 37 °C for seven days and monitored daily for the appearance of a CPE. After the incubation period, the remaining cells were detached by gentle shaking, and the fluid containing cell debris and the virus was decanted. Further, the sample was centrifuged at 134 *g* for 5 min in order to reduce potential environmental contamination, then filtered through 0.45 mm filters (Sigma-Aldrich, St. Louis, MO, USA) and stored at −80 °C until further use.

##### Nucleic Acid Extraction

Viral nucleic acid was extracted using the IndiSpin Pathogen Kit (Indical Bioscience GmbH, Leipzig, Germany) according to the manufacturer’s instructions. To ensure successful nucleic acid extraction, an external VetMax Xeno Internal Positive RNA Control (Applied Biosystems, Thermo Fisher Scientific, Waltham, MA, USA) was included in each sample.

##### MinION Sequencing

Metagenomic sequencing was performed utilizing the sequence-independent single-primer amplification (SISPA), which was previously described by [11]. The sequencing was performed on the MinION Nanopore sequencing platform. The SISPA approach uses two rounds of amplification. In Round A, there are two reaction mixes. The first one includes 11 μL of the extracted nucleic acid, 1 μL of 10 mM dNTP Mix (Invitrogen, Waltham, MA, USA), and 1 μL of a single primer FR26RV-N primer (5′ GCCGGAGCTCTGCAGATATCNNNNNN-3′) in order to obtain cDNA. The thermal profiling of the master mix is performed at 65 °C for 5 min, which is followed by cooling on ice for 1 min. The second mixture contains 4 μL of SSIV Buffer, 1 μL of SuperScript IV Reverse Transcriptase (200 U/μL), 1 μL of 100 mM DTT, and 1 μL of RNase OUT Recombinant RNase Inhibitor (Invitrogen, Waltham, USA). The obtained cDNA from the first mix and the second mix was combined and incubated at 23 °C, followed by 50 min at 50 °C, and 10 min at 80 °C, followed by the addition of 1 μL of Klenow polymerase (NEB, Ipswich, MA, USA) and further incubation for 60 min at 37 °C and then for 10 min at 75 °C.

Round B included the amplification of the Round A cDNA with the FR20RV primer (5′-GCCGGAGCTCTGCAGATATC-3′). The mix included 25 µL of PfuUltra II Hotstart PCR Master Mix (Agilent Technologies, Santa Clara, CA, USA), 1 µL of 10 µM primer, 21 µL of RNase-free water, and 3 µL of the obtained cDNA. The temperature profile included initial activation at 98 °C for 30 s, followed by 25–35 cycles of denaturation at 98 °C for 10 s, annealing at 65 °C for 30 s, and extension at 72 °C for 40 s, with a final extension at 72 °C for 2 min. The obtained products were quantified with the Qubit dsDNA High Sensitivity Assay (Thermo Fisher Scientific, Waltham, MA, USA) using the Qubit 4 fluorometer. Sequencing was performed on a MinION Mk1-c device using the R 10.4.1. flow cell and the Rapid PCR Barcoding Kit 24 V14 (SQK-RPB114.24) according to the manufacturer’s instructions.

Sequencing lasted for 12 h, the base-calling was performed with Guppy v. 24.02.16 in MinKNOW GUI—v. 5.9.18., and the initial quality score was set to 9. SISPA primers were removed, and the obtained reads were mapped using the following tools: minmap2 v2.24-r1122 (https://github.com/lh3/minimap2/releases (accessed on 10 July 2024)) was used for mapping the sequence to the reference genome, and then samtools 1.19.2 (https://github.com/samtools/samtools/releases/ (accessed on 10 July 2024)) was used for checking and indexing the obtained file, and at the end, iVar version 1.4.2 (https://github.com/andersen-lab/ivar (accessed on 19 July 2024)) was used for the creation of the consensus sequence. The reads were mapped to strains described in a previous study [12]. The obtained consensus sequences were further annotated in the Geneious Prime software (Geneious Prime 2024.0 - Geneious R9.1 - Prime 2019.0) (Dotmatics, Boston, MA, USA), and submitted to the NCBI.

### 2.2. Extraneous Agent Detection in MSV and MCL

#### 2.2.1. Cytopathic Virus Isolation Test for MSV and MCL and Hemagglutination Tests

Lyophilized MSV was reconstituted with 1 mL of sterile distilled water and after a short period of vortexing, MSV was inoculated in a cell line. MCL was prepared by triple freezing and thawing cycles, centrifuged for 5 min at 134 *g* to remove cellular debris, and inoculated on confluent monolayers of cell lines in 2 flasks of 75 cm^2^. One flask of each cell line served as a negative cell line control.

The MSV and MCL were inoculated on a confluent monolayer of the MDBK (CCLV-RIE 15), MDCK (CCLV-RIE 83), PK-15 (CCLV-RIE 56-1), VERO (CCLV-RIE 15), and BHK21 (CCLV-RIE 164) cell lines (procured from Friedrich Loeffler Institute, Department of Experimental Animal Facilities and Biorisk Management, Collection of cell lines in Veterinary Medicine (CCLV)), using DMEM (Gibco, Grand Island, NY, USA) supplemented with 10% heat-inactivated fetal bovine serum (Gibco, Grand Island, NY, USA). The inoculated cell cultures were incubated at 37 °C for seven days and monitored daily for the appearance of a cytopathic effect (CPE). After the 7 days, inoculated monolayers were frozen and thawed three times then inoculated again on fresh cell lines. Procedures were repeated 4 times and virus isolation was monitored in 4 passages of 7 days each. Hemagglutination tests of MVS and MCL were performed with all 4 passages after virus isolation.

A hemagglutination test was performed in 96-well plates with a V bottom. A volume of 25 μL of PBS solution was added to each well in two defined rows (well 2 to well 12 within row), followed by addition of 50 μL of appropriate sample of MSV and MCL to the first wells of each row; then, serial double dilutions of the starting 25 μL of volume were made from well 1 to 12 of the given rows, and eventually, the final volume of 25 μL from well 12 of each row was discarded. The plate was allowed to stand at room temperature (18 °C–24 °C) for 30 min (+/− 2 min). The next step included the addition of 50 μL of 1% avian red blood cells into all wells and the plate was allowed to stand at room temperature (18 °C–24 °C) for the next 45 min (+/− 2 min), until it was ready for results estimation. Sedimentation of erythrocytes in one point at the well bottom is a sign of a negative reaction, i.e., the absence of hemagglutination. The appearance of a net built of erythrocytes on the walls of the well without sedimentation on the wells’ bottom is a sign of positive hemagglutination.

#### 2.2.2. Fluorescent Antibody Test (FAT) Rabies Virus Detection in MSV and MCL

The absence of rabies virus was tested because the vaccine producer is working on the development of a rabies vaccine, so we thought it would be appropriate to perform this test as well. MSV and MCL prepared as previously described were simultaneously inoculated into flasks, and on a monolayer of cell line BKH 21 in 96-well microplates for rabies virus isolation. The last column of wells was inoculated with positive and negative control for rabies. Each passage of inoculated MSV and MCL was tested for the presence of rabies virus by direct immunofluorescence. After incubation, the microplates were washed, dried, fixed, and stained with FITC conjugate monoclonal anti-rabies antibodies manufactured by SIFIN (Berlin GmBH-Germany). The conjugate working solution was directly applied to the wells containing fixed cells in a volume of 50 μL. After the conjugate addition, microplates were incubated for 30 min at 37 °C in a dark humid chamber. All the wells were washed 2 times with PBS pH 7.2 for 5 min, and finally washed with distilled water. Cell monolayers in the microplate wells were observed under a fluorescent microscope in order to prove the presence or absence of rabies virus in the tested material. A direct immunofluorescence test was performed after each of the four passages of the test material, MSV and MCL. The appearance of green fluorescent granules in the inoculated cells was a sign of a positive result.

#### 2.2.3. Nucleic Acid Extraction and PCR Tests

Viral nucleic acid was extracted using the IndiSpin Pathogen Kit (Indical Bioscience GmbH, Leipzig, Germany) according to the manufacturer’s instructions. Nucleic acid extraction was performed using the automatic extraction device QIA cube Connect (produced by Qiagen GmbH Germany) according to the operation manual. QIAGEN one step RT-PCR (Qiagen, GmbH, Germany), Luna Universal Probe qPCR master mix (New England, BioLabs, Ipswich, MA, USA)), Luna Univeral Probe One Step RT-PCR (New England BioLabs, Ipswich, MA, USA), and OneTaq HotStart 2X Master Mix with Buffer (produced by New England Biolabs, USA) and the primers shown in Table 2 were used for specific genome amplifications.

### 2.3. Sterility Test

A sterility test was performed using the direct inoculation test [22] and culture method for *Mycoplasma* spp. [23]. The sterility test was performed according to the instructions of EUROPEAN PHARMACOPOEIA 10.0, monograph 2.6.1 sterility test, following the rule given in Table 2.6.1.-2: “Minimum quantity to be used for each medium”. Master seed virus was aliquoted in containers of 5 mL and 2.5 mL of MSV was inoculated in every culture medium so that the inoculum did not exceed 10% of the total volume. The sterility test was performed with 10 MVS containers each (10 replicates). Incubation of the inoculated media was not for less than 14 days. Culture media were controlled several times over the 14 days. If no evidence of microbial growth was found, MVS complied with the test for sterility. Testing for the presence of mycoplasmas was performed by the inoculation of 5 mL of MVS in 50 mL of Hayflick liquid media and incubated for 21 days. An MVS sample volume of 0.2 mL was inoculated onto a solid medium (brain heart infusion agar) and incubated for 14 days. Every 3 days, new solid media were inoculated with liquid media (subculture of liquid inoculated media). All solid media and liquid media must remain clear and free of signs of bacterial growth and the presence of mycoplasmas till the end of the *Mycoplasma* spp. test.

### 2.4. Safety Test

#### 2.4.1. Safety Test in Sheep

Safety trials on sheep were performed at the Faculty of Veterinary Medicine, University of Belgrade, Serbia. Three groups of 8 lambs serologically negative for BTV were selected to test the safety of a single-dose and double-dose candidate vaccine application. The lambs were 4 to 6 months old. The first group, A, represented the control group. The second group, B, included 8 lambs that received 1 mL of candidate vaccine (one dose) and were revaccinated with another 1 mL of the vaccine (one dose) after 21 days. The lambs in the third group, C, received 2 mL of candidate vaccine (double dose) and subsequently were revaccinated with another double dose of the vaccine (2 mL of vaccine) after 21 days. The first vaccine dose was injected subcutaneously (s.c.) in the left side of the neck, while the revaccination was performed subcutaneously in the area of the right side of the neck. All applications were given in the middle third of the neck. The control A group received 1 mL of a sterile saline solution on the same dates when the vaccines were administered to the animals in groups B and C. General and local side effects were monitored daily from the first application day up to 21 days after the second application.

#### 2.4.2. Safety Test in Pregnant Ewes

Two groups of 8 pregnant ewes of the Württemberg breed were selected for safety tests. All ewes were in the second half of pregnancy (at the end of the third month of pregnancy), which was confirmed by ultrasonography. Upon arrival at the facilities, the sheep were in good general health. The sheep were left in quarantine for seven days, during which they adjusted to the local conditions. The first group (group A) of 8 pregnant ewes received the single dose of vaccine and this was boosted after 21 days with the second single dose of 1 mL. All applications were given in the middle third of the neck, subcutaneously (s.c.). The 8 pregnant ewes of control group B received a saline solution at a dose of 1 mL in the same interval of 21 days. General and local side effects at the application site were monitored until the end of pregnancy and during parturition, as well as later, in case of an impact on the vitality of the ewes and offspring.

### 2.5. Efficacy Test

Vaccine efficacy was tested in two trials. Trial 1 was detection of seroconversion in the sera of vaccinated and revaccinated sheep by ELISA. Trial 1 was performed at the Institute of Veterinary Medicine of Serbia. The trial 2 challenge test of the vaccinated animals was performed at “Hungarian company” Prophyl “LTD Immunolab”.

#### 2.5.1. Efficacy Test, Trial No. 1

The first laboratory efficacy test served to estimate the detection of specific antibodies against BTV in vaccinated sheep, and to assess the onset of immunity and duration of immunity. The first group of 8 healthy individuals aged between 6 months and 1 year were vaccinated in the middle third of the neck subcutaneously (s.c.) and revaccinated with the candidate vaccine against BTV4 in doses of 1 mL. A control group of 8 sheep of the same age received sterile saline solution at the same volume and the same time interval. ELISA tests for specific antibodies against BT virus were performed at the Institute of Veterinary Medicine of Serbia (NRL for bluetongue disease). Serology tests were repeated up to day 382 post-immunization using the ELISA “INGEZIM BTV DR 12. BTV.K.O” produced by Ingenasa (Spain). All tests were performed according to the producer’s instructions for the use of the ELISA. ELISA was prepared on the principle of a double recognition test to detect the presence of IgG antibodies against BTV. The test is valid if the OD positive control is higher than 0.8 and the negative control is lower than the cut-off value. The cut-off is 0.15 × OD for the positive control. A sample is considered positive if its OD value is greater than the cut-off (15%).

#### 2.5.2. Efficacy Test, Trial No. 2

A challenge test was carried out by the “Hungarian company” Prophyl “LTD Immunolab”. Twenty lambs that were 6–8 months old were included in the challenge test. Ten lambs (group 1) were in the control group that received a saline solution and ten lambs (group 2) were vaccinated on day 0 of the experiment with novel candidate vaccine against BTV4 with single dose of 1 mL. The application was performed subcutaneously in the middle third of the neck on the left side. After 21 days, ten vaccinated lambs in group 2 received a second dose of the vaccine, and control group 1 received saline solution in the same way and at the same volume. On day 42, all lambs were challenged with 0.5 mL of pathogen strain BTV4 subcutaneously at a dose of 6.50 log_10_ TCID_50_. All lambs were observed until 63 days after the beginning of the trial (21 days after the challenge). All 20 lambs were monitored daily after the challenge for the presence of clinical signs of bluetongue disease such as inappetence, facial edema, hyperemia of the gums, nasal discharge, and laboured breathing.

The number of animals used for safety and efficacy tests is shown in Table 3.

## 3. Results and Discussion

### 3.1. Results of BTV4 Characterization

The genome of BTV4 was confirmed in MSV by real-time RT-qPCR. High-quality sequences were obtained for all segments besides segments 6 and 7. The obtained sequences were deposited in the NCBI under the following accession numbers: PP982459-PP982466. The identity of the sequenced segments was compared with sequences from the NCBI (Table 4).

Since guided assembly using a reference genome was employed in the analysis, there is a possibility of introducing biases, which may result in missing novel sequences, insertions, or structural variants that are absent in the reference. It is also important to consider that poor alignment in divergent or repetitive regions can lead to misassemblies or low coverage. Furthermore, the accuracy of the assembly is directly dependent on the quality and completeness of the reference genome.

### 3.2. Results of Extraneous Agent Detection in MSV and MCL

MSV was positive only for the presence of BTV, causing a cytopathogenic effect on the BHK21 cell line (a cell line susceptible to BT virus), which was confirmed later by a real-time RT-PCR test. After four passages of virus isolation on the other test cell lines, no CPE was observed. The MCL gave negative hemagglutination test results after each of four passages. All PCR tests for the detection of the genomes of non-cytopathogenic viruses and direct immunofluorescence for the detection of rabies virus were negative. There was no obvious contamination with other viruses detected in these analyses.

MSV passed the criteria considering that the sterility test for the presence of bacteria and the test for the presence of *Mycoplasma* spp. gave negative results.

The results of the master seed test gave satisfactory results. The complete absence of external virus agents from MSV as well as its sterility are the basic prerequisites for the use of MSV in the production of vaccines. The presence of any external virus agents other than the vaccine virus produced is not allowed in such a master seed that could be used for production purposes [24].

The MCL was negative for the presence of cytopathogenic viruses tested on five cell lines, MDBK, BHK21, MDCK, VERO, and PK-15, during four consecutive passages, lasting 7 days each. The MCL was also negative for the presence of hemagglutinating viruses, rabies virus, and bovine viral diarrhea virus. The master cell line was sterile according the findings of the direct inoculation test and culture method for *Mycoplasma* spp.

The tests for the presence of external agents and the sterility of the master cell line were passed and gave satisfactory results. The absence of external agents in the cell line is a prerequisite for vaccine production, and the tested BHK 21 clone 13 meets all the conditions required, based on the test results [24].

### 3.3. Safety Test Results

After the double-blind application of 1 mL of saline solution, none of the eight lambs in the control group A showed changes in the application site. The side effect results (local reaction on the application site) for group B (vaccination and revaccination with a single dose) and group C (vaccination and revaccination with a double dose) are presented in Table 4. The results for control group A are not presented in Table 5 because there were no changes in the application site throughout the experiment.

There were no changes at the application site after the administration of the first single dose of the candidate vaccine in the eight lambs in group B. After revaccination with 1 mL of the candidate vaccine in eight lambs of group B, a swollen area with a size of 1 cm in diameter appeared in six out of the eight (75%) vaccinated lambs, and spontaneously subsided in a few days without consequences on the general state of health. Two vaccinated lambs in group B (25%) had a swollen area with a size less than 1 cm in diameter.

After the administration of the first double dose (2 mL) of the candidate vaccine in eight lambs in group C, no changes at the application site were noticed. After the second administration of 2 mL of the candidate vaccine in eight lambs in the same group, local swelling with sizes of 3–5 cm in diameter appeared at the application site in five lambs (62.5%), while local swelling with a size of 1 cm in diameter appeared in three lambs (37.5%). All swelling that appeared in the lambs in group C spontaneously subsided until 42 days after the booster without treatment and consequences. There were no side effects or general disorder of the health status.

Tests of the efficacy and safety of the candidate vaccine after application were performed in accordance with the regulations on the safety of inactivated vaccines [24,25]. The safety of each inactivated vaccine must be tested with the administration of a single dose and a double dose. If the manufacturer states that the vaccine is to be applied twice with one dose within a certain time interval, the safety of the applied vaccine must be tested after two administrations of a double dose in the same prescribed time intervals. The safety has been tested by applying the recommended method of vaccine application (subcutaneous administration, s.c.) [24]. The results of the vaccination side effects gave satisfactory results and are similar or milder compared to those occurring after the application of already registered vaccines against BTV [26,27].

Safety tests of a novel vaccine against BTV4 should be performed on calves and kids as well, bearing in mind that the tests should be performed in the youngest age category of all the indicated animal species for the vaccine to ensure effectiveness in the worst-case scenario.

No changes in the general health status of the vaccinated sheep in the control and vaccinated groups during pregnancy and after parturition were noticed. No differences between the control and the experimental groups were detected. All 16 pregnant ewes, 8 in the experimental group (group A) and 8 ewes in the control group (group B), went through parturition without difficulties. All the lambs, the ones originating from the pregnant ewes from the experimental group and the ones from the control group, were vital, and no negative impact of the candidate vaccine on the health of the ewes during pregnancy and as newborn lambs was detected.

The experiment gave very good safety results for the candidate vaccine for its use in sheep during the second half of pregnancy. So far, the safety results in pregnant sheep are promising. Similar experiments should be performed on pregnant heifers and, if necessary, on pregnant goats also. Parallel testing on other indicated species would complete the results of the candidate vaccine safety since three animal species are indicated for vaccination against BTV.

### 3.4. Efficacy Test Results

#### 3.4.1. Efficacy Results of Humoral Response by ELISA Test

Eight sheep in control group A remained seronegative throughout the study period. The results of the ELISA tests for the detection of specific antibodies against BTV indicate a high degree of vaccine efficacy after the vaccination of the sheep in group B. The onset of immunity was 28 days after vaccination or earlier. In as many as 87.5% of the vaccinated animals were antibodies against BTV detected 4 weeks after revaccination. The results of the ELISA tests are presented in Table 6. The ELISA confirmed the presence of anti-BTV antibodies up to day 382 post-immunization, i.e., in seven out of eight vaccinated sheep within group B. After 42 days post-vaccination, all the vaccinated sheep from group B had specific antibodies against BTV. The sera of the vaccinated animals were finally tested on day 382 after vaccination. All eight sheep from the control group that received the placebo remained negative for the presence of specific antibodies against BTV throughout the testing period.

The laboratory serological tests on the humoral immunity in the vaccinated sheep gave quite good results. A similar vaccination test and serological tests for the presence of specific antibodies against BTV should be performed on large ruminants, in the youngest age category, with the lowest potency of novel vaccine (6.50 log_10_ TCID_50_/_mL_ before inactivation), as large ruminants are also indicated for vaccination.

**Figure 1:**
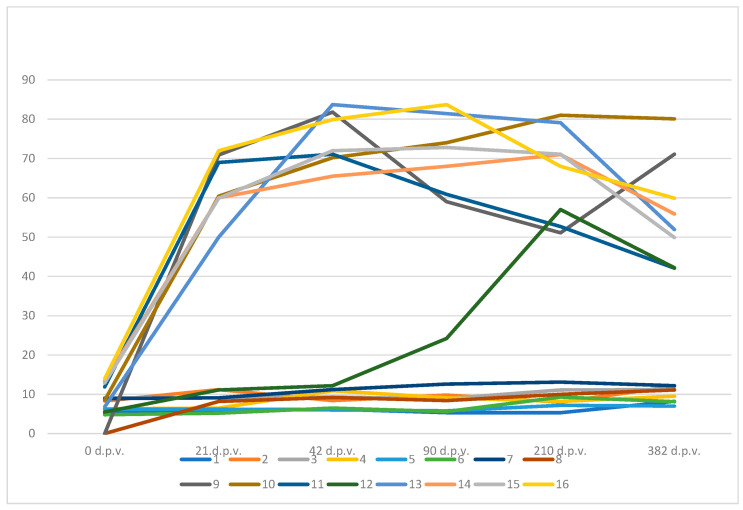
Peak level of OD% values in control and vaccinated sheep. d.p.v.—post-vaccination day, 1-8 control sheep, 9–16 vaccinated sheep.

A timeline graph for the ELISA data comparison of the peak level is presented in Figure 1.

From Figure 1, it can be concluded that all the sheep from control groups 1–8 remained negative throughout the duration of the test. The vaccinated sheep had a peak humoral immune response at 42 d.p.v. (four sheep), 90 d.p.v. (one sheep), and 210 days after vaccination (three sheep). One vaccinated sheep (No. 12) responded with a slower humoral immune response to the vaccine and its peak immune response was 210 d.p.v. The average value of the peak humoral immune response in the vaccinated sheep was 155 days after vaccination.

The results of the ELISA test are in agreement with the results of Zhugunissov et al. [28]. They tested the immunity against BTV4 from a polyvalent vaccine and obtained positive results for the presence of specific BTV4 antibodies as late as 360 days after vaccination. The trials of our vaccine confirmed the presence of specific antibodies up to 382 days after vaccination in all the vaccinated individuals. During the examination of the antibodies to BTV4 using ELISA in the individuals vaccinated with a vaccine against LSD and BTV4, El Sadeky et al. [29] confirmed the presence of specific antibodies against BTV4 in 9 out of 10 vaccinated sheep, i.e., 90%, at 360 d.p.v. Our examination confirmed the presence of BTV4 Ab in 100% of the vaccinated individuals 382 d.p.v. with the candidate vaccine.

#### 3.4.2. Results of Challenge Test

After the challenge test, all 10 sheep from the blind control group (C1-C10) developed bluetongue disease and showed moderate and typical clinical signs such as inappetence, facial edema, hyperemia of the gums, nasal discharge, and laboured breathing. A proportion of 4 of the 10 sheep (40%) that received two doses of the candidate vaccine BTV4 (V1–V10) did not show clinical signs typical for bluetongue disease. The other 6 out of 10 vaccinated individuals (60%) from the same group showed barely detectable clinical signs of bluetongue disease in the form of mild inappetence, facial edema, and nasal discharge, which disappeared within 48 to 72 h without treatment. The results are shown in Table 7.

The challenge test showed that the new vaccine against BTV protects vaccinated individuals from bluetongue disease or reduces the occurrence and duration of clinical signs of bluetongue disease. It would be helpful if efficacy tests were performed in future trials with the least potent vaccine lot on the youngest prescribed age categories of all species indicated for vaccination. In this case, the worst-case scenario would be fulfilled as a prerequisite for vaccine licensing. This would provide a guarantee that every other batch of vaccine would induce at least the same or a higher level of immunity as the least potent vaccine lot [30].

### 3.5. Duration of Immunity Against Infection from Candidate Vaccine Against BTV4

According to the results of the ELISA for the eight vaccinated sheep (group B) in Table 6, the immunity against BTV lasts at least 382 days after revaccination. Based on the results obtained from the ELISA, the manufacturer declared that the duration of immunity lasts until at least one year after vaccination. Montanid ISA 61 VG proved to be a good adjuvant considering the results obtained for the duration of immunity in the vaccinated and revaccinated individuals provided by the new vaccine.

The results of the serology trials on the duration of immunity are promising so far. It is necessary to perform a challenge test on vaccinated sheep and large ruminants after 12 months to justify the claim that the immunity provided lasts a minimum of one year after vaccination with the vaccine close to its expiration date [30]. In order to complete vaccine documentation, the vaccine should be tested on the youngest category indicated for the vaccination of calves and kids [24]. The duration of the presence of antibodies in serum must be at least 3 months longer than the declared duration of immunity from the manufacturer.

## 4. Conclusions

The preliminary results of the candidate vaccine against bluetongue disease serotype 4 have shown satisfactory results in the parameters tested so far.

The sterility of MSV and MCL was confirmed during this trial as well as the absence of external agents.

The safety test of the novel vaccine after its application gave very good results without the occurrence of general adverse effects on the health of the vaccinated lambs. The side effects were limited to a local reaction at the application site that subsided after a few weeks without treatment or therapy.

The efficacy of the tested candidate vaccine against BTV is at a satisfactory level, taking into account the results of the humoral response of the vaccinated sheep, as well as the results of the challenge test, which clarified that the candidate vaccine protects (40% of vaccinated sheep) against infection, or at least significantly reduces the intensity and duration of clinical signs (60%) after the vaccination of lambs.

The immunity lasted for at least one year.

Taking everything into account, including the current preliminary results of the purity, onset, and duration of the immunity, together with the results of the safety and efficacy tests, it is justified to be optimistic that a new vaccine against bluetongue virus serotype 4 is on the horizon.

## Figures and Tables

**Table 1:** Composition of examined candidate novel vaccine against BTV4.

Substance	Quantity in 1 mL	Function
Inactivated BTV4 4/LP/2016	≥6.5log_10_TCID_50_	Active substance
Montanide ISA 61VG	0.6 mL	Adjuvant
Thiomersal	0.05–0.1 mg	Preservative

**Table 2:** List of primers for PCR methods used in tests for external agents of MSV and MCL.

Virus	Method	Primers	References
bovine viral diarrhea	RT-qPCR	GRAGTCGTCARTGGTTCGAC	[13]
		TCAACTCCATGTGCCATGTAC	
		TGCYAYGTGGACGAGGGCATGC	
bovine ephemeral fever virus	PCR	ATGTTCAAGGTCCTCATAATTACC	[14]
		TAATGATCAAAGAACCTATCATCAC	
bovine leukemia virus	RT-PCR	CTTTGTGTGCCAAGTCTCCCAGATACA	[15]
		CCAACATATAGCACAGTCTGGGAAGGC	
parapoxvirus	PCR	TCCCTGAAGCCCTATTATTTTTGT	[16]
		GCTTGCGGGCGTTCGGACCTTC	
bovine rotavirus	RT-PCR	GGCTTTAAAAGAGAGAATTTCCGTTTGG	[17]
		GGTCACATCATACAACTCTAATCT	
tick-borne encephalitis virus	RT-PCR	GCGTTTGCT(C,T)CGGA	[18]
		CTCTTTCGACACTCGTCGAGG	
border disease virus	RT-PCR	TCGTGGTGAGATCCCTGA	[19]
		GCAGAGATTTTTTATACTAGCCTATRC	
ovine progressive pneumonia virus	RT-qPCR	GGATACCCCGAGCTCAAAG	[20]
		TTYAAKGCCCAYAGACARTT	
		TCTGTCAAGGTCTCCTTCCCG	
caprine arthritis encephalitis virus	RT-qPCR	TCTGTCAAGTKCTCCCCTCTG	[20]
		GGGAAAAGGGATTATCCTGAG	
		GTTTTAAGGCACCAYAAACAATTTC	
sheep goat poxvirus	RT-qPCR	AAATGAAACCAATGGATGGGATA	[21]
		AAAACGGTATATGGAATAGAGTTGGAA	
		TGGCTCATAGATTTCCT	
bluetongue virus	RT-qPCR	TGGAYAAAGCRATGTCAAA3	[9]
		ACRTCATCACGAAACGCTTC	
		ARGCTGCATTCGCATCGTACGC	

**Table 3:** Number of sheep used for safety and efficiency tests.

EXPERIMENT	Number of Sheep Used for Safety and Efficacy Tests		
	Control Group	Vaccinated Sheep with 2 Doses	Vaccinated Sheep with 1 Dose
Safety test in sheep	8	8	8
Safety test in pregnant ewes	8	8	0
Efficacy test 1	8	8	0
Efficacy test 2	10	10	0

**Table 4:** Identity of nucleotide sequenced segments compared with strains from the NCBI.

Segment	Accession Number (NCBI)	Highest Identity (%)	Reference Sequences	Locations
1	PP982459	99.8%	MG944817.1, MT879211.1	France, Greece
2	PP982460	99.8%	MG944818.1, OP186407.1	France, Kosovo*
3	PP982461	99.7%	MG944819.1, OP186418.1	France, Kosovo*
4	PP982462	99.9%	OP186409.1	Kosovo*
5	PP982463	99.8%	OP186410.1, OP186400.1	Kosovo*
6	N/A	N/A	N/A	N/A
7	N/A	N/A	N/A	N/A
8	PP982464	99.9%	OP186423.1	Kosovo*
9	PP982465	99.6%	OP186424.1	Kosovo*
10	PP982466	99.8%	MG944826.1	France

**Table 5:** Side effects (local reaction on the application site) of vaccination after the vaccination and revaccination with 1 mL (single dose) and 2 mL (double dose).

Sheep No	Swelling Diameter Size on the Application Site			
	Group B		Group C	
	Vacc 1 mL	Vacc. 1 mL + Revacc. 1 mL	Vacc 2 mL	Vacc. 2 mL + Revacc. 2 mL
1	No reaction	1 cm	No reaction	3–5 cm
2	No reaction	1 cm	No reaction	1 cm
3	No reaction	1 cm	No reaction	3–5 cm
4	No reaction	1 cm	No reaction	1 cm
5	No reaction	1 cm	No reaction	3–5 cm
6	No reaction	0.5 cm	No reaction	3–5 cm
7	No reaction	0.5 cm	No reaction	3–5 cm
8	No reaction	1 cm	No reaction	1 cm

**Table 6:** ELISA test results for the presence of specific antibodies against BTV in sheep sera.

Sheep	Control Group A of Sheep No 1–8 % of OD Values								Vaccinated Group B of Sheep No 9–16 % of OD Values							
Sheep No	1	2	3	4	5	6	7	8	9	10	11	12	13	14	15	16
dpv																
0 dpv	5.2	8.3	9.1	6.3	6.2	4.8	8.9	9.9	9.2	8.5	11.9	5.5	6.8	13.4	12.9	14.0
21 dpv	6.1	11.2	9.1	6.5	6.2	5.2	9.1	8.2	70.9	60.4	69.0	11.1	50.0	60	59.9	72
42 dpv	6.2	8.4	9.3	10.9	6.0	6.5	11.2	9.2	81.8	70.2	71.1	12.2	83.7	65.5	72	79.9
90 dpv	5.3	9.8	8.9	9.1	5.8	5.6	12.6	8.4	59	74	60.9	24.2	81.4	68	72.8	83.7
210 dpv	5.3	8.0	11.1	8.2	7.2	9.3	13.1	10.0	51.1	81	52.7	57	79.1	71	71.1	68
382 dpv	8.2	12.1	11.2	9.5	7.0	8.1	12.2	11.1	71.1	80.1	42.1	42.2	51.9	55.9	49.9	59.9

dpv: days post-vaccination with candidate inactivated vaccine against BTV4.

**Table 7:** Challenge test on control and revaccinated group of 10 individuals.

**BT signs in control group**	**C1**	**C2**	**C3**	**C4**	**C5**	**C6**	**C7**	**C8**	**C9**	**C10**
1 inappetence	3	3	3	3	3	3	3	3	3	2
2 facial edema	2	2	2	3	3	3	3	3	3	3
3 hyperemia of the gums	2	3	3	2	2	3	3	3	3	3
4 nasal discharge	2	3	3	3	3	3	3	2	3	2
5 laboured breathing	2	2	2	2	2	2	2	2	2	2
**BT signs in vaccinated group**	**V1**	**V2**	**V3**	**V4**	**V5**	**V6**	**V7**	**V8**	**V9**	**V10**
1 inappetence	0	1	0	0	1	1	1	1	1	0
2 facial edema	0	1	0	0	1	0-1	0-1	1	1	0
3 hyperemia of the gums	0	1	0	0	0-1	0-1	0-1	0-1	1	0
4 nasal discharge	0	1	0	0	1	1	1	1	1	0
5 laboured breathing	0	0-1	0	0	0	0	0	0	0	0

Legend: C (control group), V (vaccinated group), 0–1 (very mild, hardly visible sign), 1 (mild sign), 2 (moderate sign), 3 (clearly visible sign).

## Data Availability

The data can be shared up on request.
